# Family history of cancer as a risk factor for second malignancies after Hodgkin's lymphoma

**DOI:** 10.1038/sj.bjc.6604244

**Published:** 2008-02-12

**Authors:** A Andersson, G Enblad, B Tavelin, M Björkholm, J Linderoth, I Lagerlöf, M Merup, M Sender, B Malmer

**Affiliations:** 1 Department of Radiation Sciences (Oncology), Umeå University Hospital, 901 85 Umeå, Sweden; 2 Department of Oncology, Radiology and Clinical immunology, Section of Oncology, Uppsala University, 751 85 Uppsala, Sweden; 3 Division of Haematology, Department of Medicine, Karolinska University Hospital and Institute, 171 76 Stockholm, Sweden; 4 Department of Oncology, Lund University Hospital, 221 85 Lund, Sweden; 5 Department of Medicine, Division of Haematology, Vrinnevi Hospital, 601 82 Norrköping, Sweden; 6 Department of Hematology, Karolinska University Hospital at Huddinge, 141 86 Stockholm, Sweden; 7 Department of Oncology, Institute of Clinical Sciences, Sahglrenska University Hospital, 413 45 Göteborg, Sweden

**Keywords:** late effect, radiotherapy, second malignancy, cohort

## Abstract

This study estimated the risk of second primary malignancies after Hodgkin's lymphoma (HL) in relation to family history of cancer, age at diagnosis and latency, among 6946 patients treated for HL in Sweden in 1965–1995 identified through the Swedish Cancer Register (SCR). First-degree relatives (FDRs) to the HL patients and their malignancies were then ascertained together with their malignancies through the Multi-Generation Registry and SCR. The HL patient cohort was stratified on the number of FDRs with cancer, and standardised incidence ratios (SIRs) of developing SM were analysed. In the HL cohort, 781 SM were observed 1 year or longer after HL diagnosis. The risk for developing SM increased with the number of FDRs with cancer, SIRs being 2.26, 3.01, and 3.45 with 0, 1, or ⩾2 FDRs with cancer, respectively. Hodgkin's lymphoma long-term survivors treated at a young age with a family history of cancer carry an increased risk for developing SM and may represent a subgroup where standardised screening for the most common cancer sites could be offered in a stringent surveillance programme.

The proportion of long-term survivors following Hodgkin's lymphoma (HL) has increased since the mid-twentieth century (Devita *et al*, 1970; Jenkin *et al*, 1990; Fuchs *et al*, 2006). Long-term survivors of HL have a shorter life expectancy compared with the normal population, since they have a significantly elevated risk of developing second malignancies (SMs), heart diseases, lung diseases, and infections; SM being the commonest cause of death (Mauch, 1996; Hoppe, 1997; Swerdlow *et al*, 2000; Dores *et al*, 2002; Foss Abrahamsen *et al*, 2002; Ng *et al*, 2002; Aleman *et al*, 2003). The incidence of SM increase especially after 10 years latency and among patients treated below the age of 40 years, in which radiotherapy has been suggested as the main underlying factor. Haematological malignancies develop earlier, within the first 10 years after treatment, probably due to the chemotherapy and not radiotherapy (Kaldor *et al*, 1990). It is not established in long-term follow-up if the frequency of SM is reduced with lower doses of radiation and reduced target fields (Franklin *et al*, 2005).

In the present study, we estimated the standardised incidence ratio (SIR) for SM in the Swedish population with HL diagnosed in Sweden in 1965–1995 and in relation to family history of cancer.

## PATIENTS AND METHODS

By law since 1958, all malignancies in Sweden must be reported to the Swedish Cancer Register (SCR), at the National Board of Health and Welfare. From this register, 6946 HL cases diagnosed in 1965–1995 were identified. Early in this period, extended field irradiation, mainly mantle field, was the standard treatment. Patients received radiotherapy, chemotherapy or a combination of both. Details of disease extent, therapy or its results are not registered in the SCR, and nor on different HL subtypes. There were 2873 female and 4073 male patients, with mean age at diagnosis of 52 years. Date and cause of death were obtained by matching the cohort to the Cause of Death Register, which was updated until 31 December 2004.

In the HL cohort, 1993 were alive at the end of 2004 and among the 4953 deceased, the mean age at death was 65 years; 2311 individuals died within the first year after diagnosis. Among the 2642 who died 1 year or later after HL diagnosis, 570 (21.3 %) died with the occurrence of SM during the follow-up, with a mean age of death of 65.8 years. Characteristics of the patients are shown in Table 1. The patients were observed until 31 December 2004, time of migration from Sweden or death, whichever came first and linked to SCR for SM. The date of occurrence of a new malignancy and the site of origin were registered. The first year after HL diagnosis was omitted because of the likelihood of excess cases being due to increased surveillance (Thellenberg *et al*, 2003).

The expected number of cancer cases was calculated by multiplying the person-years for every calendar year, sex, and 5-year age group by the corresponding age-specific incidence rate in Sweden. The SIR was calculated by dividing the observed number of cases by the number expected (Breslow and Day, 1980). The risk was estimated 1–9, 10–19, and ⩾20 years after treatment of HL with a 95% confidence interval (CI).

We also investigated whether a familial history of cancer further increased the risk of SM after HL. From the Multi-Generation Registry at Statistics, Sweden, (MGR), first-degree relatives (FDRs) to our cohort of the initial 6946 HL patients were collected. First-degree relatives include parents, siblings, and children. Because the MGR includes parents for children born after 1932, full nuclear families are not available for all patients in the HL cohort. If a person died before 1961, he might be lost by follow-up (Hemminki *et al*, 2001). In this register, there were 17 858 FDRs to the HL cohort, who were then matched to the SCR to determine their cancer incidence, and hence the family history of cancer for each HL patient was then calculated. The cohort of relatives was compared with the normal population in Sweden to estimate the cancer SIRs. Finally, SIRs were calculated in the HL cohort for SM when stratified for having 0, 1, or ⩾2 relatives with cancer. The absolute excess risk was calculated as the observed number of SMs in our cohort minus that expected divided by the number of person-years at risk multiplied by 10 000.

## RESULTS

**Figure 1:**
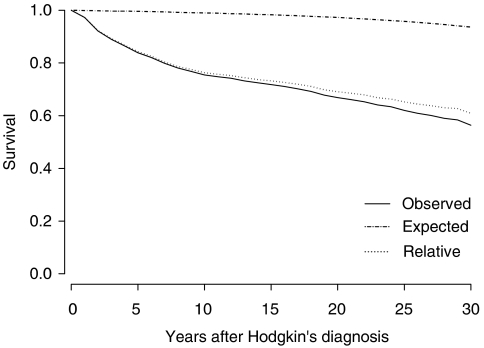
Absolute and relative survival for HL patients treated before the age of 40 years between the years of 1965 and 1995 compared with the normal population in Sweden.

Survival in HL patients treated for HL at 40 years or younger was decreased compared with the general Swedish population, and also 10 years or more after treatment (Figure 1). Even the relative survival – matched for age, sex, and year – was decreased. Of the 6946 HL patients, 4623 survived more than 1 year after diagnosis and of these 696 (15%) developed a total of 781 SMs. Solid tumours accounted for 645 (82.6%) of these and haematological malignancies for 136 (17.4%). The SIR for SM overall was significantly increased, at 2.62 (95% CI: 2.32–2.96) 10–19 years after HL diagnosis (Table 2). When stratified for age at HL diagnosis, incidence was especially increased among those diagnosed before the age of 40 years, SIR 4.34 (95% CI: 3.51–5.30).

One of the commonest SM was breast cancer with risk increased 10–19 years after HL only seen in the group treated before the age of 40 years, SIR 5.20 (95% CI: 3.39–7.62), compared with those aged 40 years or older, SIR 1.23 (95% CI: 0.56–2.34). Other common cancers overrepresented were lung, SIR 3.26 (95% CI: 2.09–4.85) and colorectal cancers, SIR 2.42 (95% CI: 1.60–3.49) (Table 2). In the cohort of 17 858 FDRs, there were 4440 parents, 4611 siblings, and 8807 children. There was no increased risk for developing cancer overall in the cohort of relatives compared with the general population, SIR 1.01 (95% CI: 0.97–1.05).

In the HL cohort, 1453 individuals (20.9%) had one or more FDRs with cancer. There was a gradually increased risk for SM with the number of FDRs with cancer, one SIR 3.01 (2.57–3.51) and two or more SIRs 3.45 (2.58–4.51) (Table 3).

The risks of breast, lung, and colorectal cancers stratified for the number of FDRs with cancer overall were calculated, initially on 0, 1, or 2 or more FDRs, but as there were no obvious trends and the numbers were small, the analyses simply considered positive or negative family history of cancer. The risk for breast cancer was increased with a positive family history of cancer, especially after >20 years follow-up, SIR 5.52 (3.32–8.62) (Table 4). For all HL patients with a family history of lung cancer, there seems to be an especially increased risk of lung cancer ⩾10 years after treatment of HL. No corresponding increased risk of breast cancer with a family history of breast cancer was observed.

## DISCUSSION

Risk of SM was especially increased in young patients with a family history of cancer and a long follow-up. This could indicate a gene–environment or environment–environment interaction, as recently proposed in other studies of cohorts exposed to ionising radiation (Flint-Richter and Sadetzki, 2007). Strengths of the present study are the mean follow-up of 12.3 years (range: 0–38 years) and that the SCR is known to be complete and nationwide, thereby excluding underreporting (Frodin *et al*, 1997; Sandblom *et al*, 2003).

Our data do not suggest that a family history of breast cancer adds to the risk of secondary breast cancer. In another study increasing doses of RT did not add to the risk of breast cancer in the presence of a positive family history of breast or ovarian cancer in first- or second-degree relatives (Hill *et al*, 2005).

A novel observation was an increased risk of lung cancer in HL patients with a family history of lung cancer, which might be due to genetic or environmental causes such as smoking habits; although details of these were not available in this study.

Genetic factors were suggested to contribute to SM risk in HL survivors, although here family history of cancer was collected using patient interviews and medical records and not systematically from a cancer register (Nichols *et al*, 1999). Only one large study has investigated the family history of cancer overall in relation to SM risk after lymphoma, including HL (Landgren *et al*, 2007). The 7476 HL cases studied from Sweden and Denmark with a shorter follow-up partly overlapped with our study. Their study showed an increased risk for breast cancer, RR 1.81 (95% CI: 1.04–3.16) in the HL subgroup with a positive family history of cancer. Whereas they estimated risks with Cox proportional hazard ratios, we used a comparison to the general population. We omitted the first year after HL diagnosis to avoid overestimating the risk due to increased surveillance. The present study indicates that cancer among FDRs gives an additional increase to SM risk among HL survivors. There was also a trend of increased risk of SM with the numbers of relatives with malignancies, although there were few with two or more relatives with cancer.

A surveillance programme for women treated with radiotherapy for HL has proposed investigation with the same standard as other groups of women at higher risk for breast cancer (Dershaw *et al*, 1992; Wolden *et al*, 2000, Diller *et al*, 2002, Faulkner and Law, 2005). An increased risk of secondary lung cancer has been reported (Metayer *et al*, 2000; Foss Abrahamsen *et al*, 2002), while chemotherapy and radiotherapy were associated with an elevated risk of lung cancer as an SM further increased by smoking habits up to 20-fold (Travis *et al*, 2002). We also found lung cancer risk increasing from 10 years after treatment, seeming to increase continuously thereafter, particularly in men (Table 2). An environmental interaction of radiotherapy and smoking can be suspected. Increased risk in the first 10 years could be due to chemotherapy or the increased numbers of computed tomography (CT) scans for detecting smaller tumours during HL follow-up. There is no good evidence that screening with CT of the lung will have any cost benefit in nonsmokers, although larger studies are necessary to address this question (Das *et al*, 2006). There is no obvious pattern of decreased SM risk after a certain number years except for breast cancer.

A drawback of the study is that the Swedish Cancer Register holds no therapy data, but studies have shown that up to 90% of patients were given radiotherapy during our study period (Glimelius *et al*, 1994, Molin *et al*, 2003). Many HL patients were probably diagnosed as nonHodgkin's lymphoma (NHL), but because most of them were older patients with a short survival, this will not affect the present results, especially those with long follow-up that are compared with the general population. It might, however, influence overall survival as prognosis is generally poorer for NHL.

The high curability of HL has increased the number of long-term survivors (Fuchs *et al*, 2006). In our cohort of 2000 HL survivors, most were treated with extended radiation field. A Swedish pilot study (unpublished) indicates that only about 60% of these patients have regular clinic visits. Women over 40 years of age participate in the general mammography screening programme. The Swedish HL Group currently offers patients who are not participating in regular clinic visits a standardised surveillance programme. The goal is to find SM at an earlier stage and thereby raise the cure rate. Mammography and ultrasound will be offered to women treated before the age of 40 years. Patients with gastrointestinal or pulmonary symptoms will be offered a radiological examination. The importance of smoking cessation is stressed and patients will be offered referral to smoking cessation programmes.

Our study indicates that SM incidence is increased among the long-term HL survivors with a family history of cancer, young age of onset, and long latency, perhaps reflecting gene–environment interactions; this could be relevant in future surveillance programmes.

## Figures and Tables

**Table 1:** Characteristics of the Hodgkin's lymphoma patients and their first-degree relatives

**Characteristics**	
Number of patients, total	6946
Male	4073
Female	2873
Mean age at diagnosis, years (range)	52 (2–100)
Mean age <40 years at diagnose, *n*	2379
<40 years at diagnosis and alive at the end of 2004, *n*	1558
Diagnosis year, range	1965–1995
Number of deceased	4953
Mean age at death, years (range)	65 (7–100)
Number of patients with follow-up ⩾1 year	4623
	
Mean follow-up (years)^a^	
All	12.3 (0–38)
Alive at the end of follow-up	19.0 (8–38)
First-degree relatives	17 858^b^
Parents	4440
Siblings	4611
Offspring	8807

a Calculated for individuals with a follow-up ⩾1 year.

b 17 798 unique individuals.

**Table 2:** Risk for second cancer overall and cancer at the most common sites, 1–9, 10–19 and 20 years or over after HL, for all ages and <40 years at HL treatment

	**Year after treatment**							
	**1–9**		**10–19**		**20** ^−∞^		**1** ^−∞^	
**Site**	**O**	**SIR (95% CI)**	**O**	**SIR (95% CI)**	**O**	**SIR (95% CI)**	**O**	**E/SIR (95% CI)**
*Treated for HL at all ages*								
Cancer overall	349	2.39 (2.14–2.65)	265	2.62 (2.32–2.96)	167	2.44 (2.08–2.84)	781	2.48 (2.30–2.65)
Breast	15	0.98 (0.55–1.61)	35	2.84 (1.98–3.95)	31	3.52 (2.39–5.00)	81	2.22 (1.76–2.76)
Colorectal	37	2.94 (2.06–4.04)	28	2.42 (1.60–3.49)	17	2.21 (1.28–3.53)	82	2.57 (2.04–3.19)
Kidney	4	0.89 (0.23–2.16)	5	1.73 (0.56–4.04)	4	2.32 (0.62–5.93)	13	1.39 (0.73–2.37)
Leukaemia^a^	38	6.61 (4.67–9.07)	10	2.67 (1.28–4.91)	9	3.57 (1.62–6.78)	57	4.75 (3.59–6.14)
Lung	31	2.83 (1.92–4.01)	24	3.26 (2.09–4.85)	27	5.40 (3.56–7.86)	82	3.59 (2.85–4.46)
NHL	36	8.59 (6.02–11.90)	26	8.34 (5.44–12.22)	14	6.67 (3.64–11.19)	76	8.08 (6.36–10.10)
Pancreas	5	1.11 (0.36–2.59)	8	3.12 (1.34–6.14)	8	5.40 (2.32–10.64)	21	2.45 (1.51–3.75)
Prostate	32	1.54 (1.05–2.17)	20	1.30 (0.79–2.00)	9	0.73 (0.33–1.38)	61	1.25 (0.95–1.61)
Skin^b^	39	6.70 (4.76–9.15)	28	5.93 (3.94–8.58)	11	3.01 (1.50–5.39)	78	5.50 (4.34–6.86)
Thyroid	5	3.98 (1.28–9.28)	8	9.99 (4.30–19.68)	2	4.99 (0.56–18.03)	15	6.10 (3.41–10.05)
								
*Treated for HL*<*age 40 years*								
Cancer overall	58	4.66 (3.54–6.03)	96	4.34 (3.51–5.30)	119	3.60 (2.98–4.31)	273	4.03 (3.56–4.53)
Breast	3	1.36 (0.27–3.97)	26	5.20 (3.39–7.62)	27	4.55 (2.99–6.61)	56	4.22 (3.19–5.48)
Colorectal	2	2.70 (0.30–9.76)	5	3.09 (0.99–7.21)	12	3.86 (1.99–6.74)	19	3.54 (2.12–5.52)
Kidney	0	—	3	5.56 (1.11–16.23)	2	2.33 (0.26–8.39)	5	3.01 (0.97–7.02)
Leukaemia^a^	17	30.91 (17.99–49.49)	6	5.13 (1.87–5.13)	1	0.88 (0.01–4.92)	24	8.42 (5.39–12.53)
Lung	2	5.26 (0.59–19.00)	2	1.68 (0.18–6.07)	22	8.91 (5.57–14.49)	26	6.44 (4.20–9.43)
NHL	8	16.67 (7.17–32.84)	10	12.5 (5.98–22.99)	10	9.62 (4.60–17.68)	28	12.02 (7.98–17.36)
Pancreas	0	—	1	3.03 (0.03–16.86)	6	9.52 (3.47–20.73)	7	6.48 (2.59–13.35)
Prostate	0	—	1	1.47 (0.01–8.18)	4	0.86 (0.23–2.21)	5	0.94 (0.30–2.18)
Skin^b^	1	4.55 (00.59–25.29)	6	12.00 (4.38–26.12)	7	7.00 (2.80–14.42)	14	8.05 (4.39–13.50)
Thyroid	2	4.88 (0.54–17.61)	6	14.29 (5.21–31.09)	2	7.69 (0.86–27.77)	10	9.01 (4.31–16.56)
								
*Treated for HL⩾ age 40 year*								
Cancer overall	291	2.18 (1.93–2.45)	169	2.14 (1.83–2.49)	48	1.36 (1.00–1.80)	508	2.05 (1.87–2.23)
Breast	12	0.92 (0.48–2.18)	9	1.23 (0.52–2.34)	4	1.48 (0.40–3.78)	25	1.08 (0.70–1.59)

CI=confidence interval; HL=Hodgkin's lymphoma; MDS=myelodysplastic syndromes; NHL=nonHodgkin's lymphoma; SIR=standardised incidence ratio.

For cancer overall and breast cancer, the risk has also been calculated among individuals treated at the age of 40 years or older.

a The group Leukaemia includes MDS, myeloma, lymphatic leukaemia, myeloic leukaemia, monocytic leukaemia, and leukaemia UNS.

b IC7 191. Malignant melanomas excluded.

**Table 3:** Risk for second cancer overall after HL at any age and before the age of 40 years stratified on the number of first-degree relatives with cancer overall

		**Number of first-degree relatives with cancer overall**					
		**0**		**1**		**2 or >2**	
**Age at treatment**	**Years, after HL**	**O**	**SIR (95% CI)**	**O**	**SIR (95% CI)**	**O**	**SIR (95% CI)**
All ages	⩾1	561	2.26 (2.08–2.45)	167	3.01 (2.57–3.51)	53	3.45 (2.58–4.51)
							
All ages	1–9	284	2.31 (2.05–2.59)	54	2.74 (2.06–3.57)	11	2.58 (1.29–4.62)
	10–19	185	2.39 (2.06–2.76)	57	2.99 (2.27–3.88)	23	4.15 (2.63–6.23)
	⩾20	92	1.92 (1.55–2.36)	56	3.37 (2.54–4.37)	19	3.41 (2.05–5.33)
							
<40 years	⩾1	141	3.78 (3.18–4.46)	101	4.35 (3.54–5.28)	39	4.35 (3.09–5.95)
							
<40 years	1–9	37	4.84 (3.41–6.68)	15	3.75 (2.10–6.19)	6	4.41 (1.61–9.60)
	10–19	47	3.85 (2.83–5.12)	35	4.69 (3.26–6.52)	14	4.91 (2.68–8.24)
	⩾20	52	2.97 (2.22–3.89)	48	4.06 (3.00–5.39)	19	3.98 (2.40–6.22)

CI=confidence interval; HL=Hodgkin's lymphoma; SIR=standardised incidence ratio.

Hodgkin diagnoses.

**Table 4:** Risk for second lung, breast, or colorectal cancers stratified, respectively, on the presence of first-degree relatives with cancer overall or first-degree relative with cancer at risk

			**Presence of first-degree relatives with cancer overall**					
			**Negative**			**Positive**		
**Age at treatment**	**Second cancer, site**	**Years, after HL**	**O**	**SIR (95% CI)**	**AER**	**O**	**SIR (95% CI)**	**AER**
All ages	Breast	⩾1	40	1.53 (1.10–2.09)	3.6	41	3.96 (2.83–5.36)	15.5
		1–9	8	0.65 (0.28–1.28)	—	7	2.31 (0.92–4.76)	4.3
		10–19	20	2.37 (1.45–3.66)	9.7	15	3.87 (2.16–6.38)	16.5
		⩾20	12	2.23 (1.15–3.90)	13.0	19	5.52 (3.32–8.62)	40.8
								
	Lung	⩾1	59	3.20 (2.44–4.13)	10.6	23	4.57 (2.89–6.86)	9.1
		1–9	25	2.69 (1.74–3.97)	7.4	6	3.55 (1.29–7.73)	4.7
		10–19	18	3.16 (1.87–4.99)	10.3	6	3.55 (1.29–7.73)	6.4
		⩾20	16	4.6 (2.64–7.51)	24.6	11	6.71 (3.34–12.00)	24.4
								
	Colorectal	⩾1	64	2.13 (1.63–2.71)	8.9	18	2.54 (1.50–4.01)	5.5
		1–9	31	2.05 (1.39–2.91)	7.5	6	2.43 (0.88–5.29)	3.8
		10–19	25	2.71 (1.75–4.01)	13.2	3	1.24 (0.25–3.64)	0.9
		⩾20	8	1.41 (0.60–2.78)	4.6	9	4.13 (1.88–7.84)	17.9

AER=absolute excess risk; CI=confidence interval; HL=Hodgkin's lymphoma; SIR=standardised incidence ratio.

Time after treatment divided into 1–9, 10–19, and 20 or over 20 years after treatment for HL. Male and female subjects combined. Treated for HL at all ages.

## References

[bib1] Aleman BM, van den Belt-Dusebout AW, Klokman WJ, Van't Veer MB, Bartelink H, van Leeuwen FE (2003) Long-term cause-specific mortality of patients treated for Hodgkin's disease. J Clin Oncol 21: 3431–3439 12885835 10.1200/JCO.2003.07.131

[bib2] Breslow NE, Day NE (1980) Statistical methods in cancer research. Volume I – The analysis of case–control studies. IARC Sci Publ 32: 5–338 7216345

[bib3] Das P, Ng AK, Earle CC, Mauch PM, Kuntz KM (2006) Computed tomography screening for lung cancer in Hodgkin's lymphoma survivors: decision analysis and cost-effectiveness analysis. Ann Oncol 17: 785–793 16500905 10.1093/annonc/mdl023

[bib4] Dershaw DD, Yahalom J, Petrek JA (1992) Breast carcinoma in women previously treated for Hodgkin disease: mammographic evaluation. Radiology 184: 421–423 1320281 10.1148/radiology.184.2.1320281

[bib5] Devita Jr VT, Serpick AA, Carbone PP (1970) Combination chemotherapy in the treatment of advanced Hodgkin's disease. Ann Intern Med 73: 881–895 5525541 10.7326/0003-4819-73-6-881

[bib6] Diller L, Medeiros Nancarrow C, Shaffer K, Matulonis U, Mauch P, Neuberg D, Tarbell NJ, Litman H, Garber J (2002) Breast cancer screening in women previously treated for Hodgkin's disease: a prospective cohort study. J Clin Oncol 20: 2085–2091 11956269 10.1200/JCO.2002.08.031

[bib7] Dores GM, Metayer C, Curtis RE, Lynch CF, Clarke EA, Glimelius B, Storm H, Pukkala E, van Leeuwen FE, Holowaty EJ, Andersson M, Wiklund T, Joensuu T, van't Veer MB, Stovall M, Gospodarowicz M, Travis LB (2002) Second malignant neoplasms among long-term survivors of Hodgkin's disease: a population-based evaluation over 25 years. J Clin Oncol 20: 3484–3494 12177110 10.1200/JCO.2002.09.038

[bib8] Faulkner K, Law J (2005) Mammographic breast cancer screening for women previously treated with high breast doses for diseases such as Hodgkin's. Radiat Prot Dosimetry 117: 330–333 16461484 10.1093/rpd/nci766

[bib9] Flint-Richter P, Sadetzki S (2007) Genetic predisposition for the development of radiation-associated meningioma: an epidemiological study. Lancet Oncol 8: 403–410 17466897 10.1016/S1470-2045(07)70107-9

[bib10] Foss Abrahamsen A, Andersen A, Nome O, Jacobsen AB, Holte H, Foss Abrahamsen J, Kvaloy S (2002) Long-term risk of second malignancy after treatment of Hodgkin's disease: the influence of treatment, age and follow-up time. Ann Oncol 13: 1786–1791 12419752 10.1093/annonc/mdf289

[bib11] Franklin JG, Paus MD, Pluetschow A, Specht L (2005) Chemotherapy, radiotherapy and combined modality for Hodgkin's disease, with emphasis on second cancer risk. Cochrane Database Syst Rev 4: CD003187 16235316 10.1002/14651858.CD003187.pub2 PMC7017637

[bib12] Frodin JE, Ericsson J, Barlow L (1997) Multiple primary malignant tumors in a national cancer registry – reliability of reporting. Acta Oncol 36: 465–469 9292741 10.3109/02841869709001300

[bib13] Fuchs M, Diehl V, Re D (2006) Current strategies and new approaches in the treatment of Hodgkin's lymphoma. Pathobiology 73: 126–140 17085957 10.1159/000095559

[bib14] Glimelius B, Kalkner M, Enblad G, Gustavsson A, Jakobsson M, Branehog I, Lenner P (1994) Treatment of early and intermediate stages of supradiaphragmatic Hodgkin's disease: the Swedish National Care Programme experience. Swedish Lymphoma Study Group. Ann Oncol 5: 809–816 7848883 10.1093/oxfordjournals.annonc.a059009

[bib15] Hemminki K, Li X, Plna K, Granstrom C, Vaittinen P (2001) The nation-wide Swedish family-cancer database – updated structure and familial rates. Acta Oncol 40: 772–777 11765074 10.1080/02841860152619214

[bib16] Hill DA, Gilbert E, Dores GM, Gospodarowicz M, van Leeuwen FE, Holowaty E, Glimelius B, Andersson M, Wiklund T, Lynch CF, Van't Veer M, Storm H, Pukkala E, Stovall M, Curtis RE, Allan JM, Boice JD, Travis LB (2005) Breast cancer risk following radiotherapy for Hodgkin lymphoma: modification by other risk factors. Blood 106: 3358–3365 16051739 10.1182/blood-2005-04-1535 PMC1895063

[bib17] Hoppe RT (1997) Hodgkin's disease: complications of therapy and excess mortality. Ann Oncol 8(Suppl 1): 115–118 9187444

[bib18] Jenkin D, Doyle J, Berry M, Blanchette V, Chan H, Doherty M, Freedman M, Greenberg M, Panzarella T, Saunders F, Sonley M, Weitzman S, Zipursky A (1990) Hodgkin's disease in children: treatment with MOPP and low-dose, extended field irradiation without laparotomy. Late results and toxicity. Med Pediatr Oncol 18: 265–272 2355885 10.1002/mpo.2950180402

[bib19] Kaldor JM, Day NE, Clarke EA, Van Leeuwen FE, Henry-Amar M, Fiorentino MV, Bell J, Pedersen D, Band P, Assouline D, Koch M, Choi W, Prior P, Blair V, Langmark F, Pompe Kirn V, Neal F, Peters D, Pfeiffer R, Karjalainen S, Cuzick J, Sutcliffe S, Somers R, Pellae-Cosset B, Pappagallo G, Frasier P, Storm H, Stovall M (1990) Leukemia following Hodgkin's disease. N Engl J Med 322: 7–13 2403650 10.1056/NEJM199001043220102

[bib20] Landgren O, Pfeiffer RM, Stewart L, Gridley G, Mellemkjaer L, Hemminki K, Goldin LR, Travis LB (2007) Risk of second malignant neoplasms among lymphoma patients with a family history of cancer. Int J Cancer 120: 1099–1102 17131330 10.1002/ijc.22414

[bib21] Mauch PM (1996) Management of early stage Hodgkin's disease: the role of radiation therapy and/or chemotherapy. Baillieres Clin Haematol 9: 531–541 8922243 10.1016/s0950-3536(96)80024-2

[bib22] Metayer C, Lynch CF, Clarke EA, Glimelius B, Storm H, Pukkala E, Joensuu T, van Leeuwen FE, van't Veer MB, Curtis RE, Holowaty EJ, Andersson M, Wiklund T, Gospodarowicz M, Travis LB (2000) Second cancers among long-term survivors of Hodgkin's disease diagnosed in childhood and adolescence. J Clin Oncol 18: 2435–2443 10856104 10.1200/JCO.2000.18.12.2435

[bib23] Molin D, Enblad G, Gustavsson A, Ekman T, Erlanson M, Haapaniemi E, Glimelius B (2003) Early and intermediate stage Hodgkin's lymphoma – report from the Swedish National Care Programme. Eur J Haematol 70: 172–180 12605661 10.1034/j.1600-0609.2003.00030.x

[bib24] Ng AK, Bernardo MP, Weller E, Backstrand KH, Silver B, Marcus KC, Tarbell NJ, Friedberg J, Canellos GP, Mauch PM (2002) Long-term survival and competing causes of death in patients with early-stage Hodgkin's disease treated at age 50 or younger. J Clin Oncol 20: 2101–2108 11956271 10.1200/JCO.2002.08.021

[bib25] Nichols KE, Levitz S, Shannon KE, Wahrer DC, Bell DW, Chang G, Hegde S, Neuberg D, Shafman T, Tarbell NJ, Mauch P, Ishioka C, Haber DA, Diller L (1999) Heterozygous germline ATM mutations do not contribute to radiation-associated malignancies after Hodgkin's disease. J Clin Oncol 17: 1259 10561187 10.1200/JCO.1999.17.4.1259

[bib26] Sandblom G, Dufmats M, Olsson M, Varenhorst E (2003) Validity of a population-based cancer register in Sweden – an assessment of data reproducibility in the South-East Region Prostate Cancer Register. Scand J Urol Nephrol 37: 112–119 12745718 10.1080/00365590310008839

[bib27] Swerdlow AJ, Barber JA, Hudson GV, Cunningham D, Gupta RK, Hancock BW, Horwich A, Lister TA, Linch DC (2000) Risk of second malignancy after Hodgkin's disease in a collaborative British cohort: the relation to age at treatment. J Clin Oncol 18: 498–509 10653865 10.1200/JCO.2000.18.3.498

[bib28] Thellenberg C, Malmer B, Tavelin B, Gronberg H (2003) Second primary cancers in men with prostate cancer: an increased risk of male breast cancer. J Urol 169: 1345–1348 12629357 10.1097/01.ju.0000056706.88960.7c

[bib29] Travis LB, Gospodarowicz M, Curtis RE, Clarke EA, Andersson M, Glimelius B, Joensuu T, Lynch CF, van Leeuwen FE, Holowaty E, Storm H, Glimelius I, Pukkala E, Stovall M, Fraumeni Jr JF, Boice Jr JD, Gilbert E (2002) Lung cancer following chemotherapy and radiotherapy for Hodgkin's disease. J Natl Cancer Inst 94: 182–192 11830608 10.1093/jnci/94.3.182

[bib30] Wolden SL, Hancock SL, Carlson RW, Goffinet DR, Jeffrey SS, Hoppe RT (2000) Management of breast cancer after Hodgkin's disease. J Clin Oncol 18: 765–772 10673517 10.1200/JCO.2000.18.4.765

